# 2D materials readiness for the transistor performance breakthrough

**DOI:** 10.1016/j.isci.2023.106673

**Published:** 2023-04-19

**Authors:** Qing Zhang, Chunsen Liu, Peng Zhou

**Affiliations:** 1 State Key Laboratory of ASIC and System, School of Microelectronics, Fudan University, Shanghai 200433, China; 2 Frontier Institute of Chip and System, Fudan University, Shanghai 200433, China

**Keywords:** Applied sciences, Materials science, Nanomaterials

## Abstract

As the size of the transistor scales down, this strategy has confronted challenges because of the fundamental limits of silicon materials. Besides, more and more energy and time are consumed by the data transmission out of transistor computing because of the speed mismatching between the computing and memory. To meet the energy efficiency demands of big data computing, the transistor should have a smaller feature size and store data faster to overcome the energy burden of computing and data transfer. Electron transport in two-dimensional (2D) materials is constrained within a 2D plane and different materials are assembled by the van der Waals force. Owning to the atomic thickness and dangling-bond-free surface, 2D materials have demonstrated advantages in transistor scaling-down and heterogeneous structure innovation. In this review, from the performance breakthrough of 2D transistors, we discuss the opportunities, progress and challenges of 2D materials in transistor applications.

## Introduction

The continuous scaling down of the transistor size drives the performance booming of integrated circuits. However, with the technology node of the transistor below 5 nm, the state-of-the-art transistor structure (Fin field-effect-transistor, FinFET) becomes unsustainable for performance enhancement. The scaling down of FinFET needs taller and thinner Fin channels. The tall Fin and aggressive Fin pitch will lead to sidewalls damages to Fins^1^ and the mobility of the silicon material with a thickness below 3 nm will also be significantly degraded.^2^^,^^3^ What’s more, the lattice mismatch issues between different bulk materials have also limited the development of novel heterostructure transistors.^4^ Heterostructure transistors with optimized electrical band structure can better support device structure innovation and performance breakthroughs. For example, heterostructure can advance the band-to-band tunneling efficiency in the tunneling FET (TFET) and enables steeper subthreshold swing (SS). Therefore, a material system with good electrical characteristics in ultrathin thickness and owning flexible heterostructure integration ability will be favorable for breaking the transistor performance bottleneck.

Except for the transistor scaling down issue, the energy and time consumption of the system is also limited by the data shuttle efficiency.^2^ The most widely used computer architecture is the von Neumann architecture where the computing and memory modules are physically separated. The data shuttle rate between the computing module and the memory module is limited by this architecture, leading to large energy and time consumption when the processing data is big, even larger than the consumption of the computing itself.^5^ The volume of high-speed volatile transistor memory is too small to memorize all the data, whereas the high-density non-volatile transistor memory is too slow to timely stored or recall data. Although the different volatile memory chips can be connected through silicon via interconnects to accelerate the data transfer process,^6^ the limited connection density of silicon via is not adequate for highly energy-efficient computing.^7^^,^^8^ An ultrafast non-volatile transistor memory technology that supports high-density integration is needed.

2D materials are covalently bonded in a plane with a dangling-bond-free surface, and a single layer (thickness below 1 nm) can be easily achieved from a bulk crystal.^9^ Because the neighboring layer in 2D materials is connected only by a weak van der Waals (vdW) force, the electrical characteristics of 2D materials will not degrade when their thickness thins to monolayer. Benefiting from this unique characteristic, the electron transportation is tied to a thin channel^10^ which means that the space charge region in the drain/source will not expand along the channel direction. This transportation process is similar to the silicon-on-insulator technology and hence the 2D materials transistor can be immune short channel effect. What’s more, the different 2D materials can be assembled to form various heterostructures by a van der Waals stacking technology because of their dangling-bond-free surface. The 2D materials are a big material system and can provide enough options for the device structure and function design. It is predicted that over one thousand kinds of 2D materials can be easily exfoliated and various 2D materials demonstrate abundant properties,^11^ such as electronic,^12^^,^^13^^,^^14^ photoelectric,^15^ magnetic^16^ and ferroelectrics^17^^,^^18^ properties. With good electrical performance in small feature sizes and flexible heterostructure constructing ability, 2D materials are possible to solve the performance bottlenecks of the transistor.

In this article, we review the performance breakthrough of 2D transistors. The discussion of the 2D transistors is focused on the scaling down issue, contact optimization, ultrathin gate oxide, steep subthreshold swing (SS), and ultrafast charge storage speed. Finally, we discuss the challenges that hinder 2D transistors from large-scale applications.

## Discussion and results

2D materials are different from silicon in the structure, which is covalently bonded in the plane and adjacent layers are linked only by the vdW force. Therefore, the basic fabrication processes and the performance modulation methods of the 2D transistors are not like the silicon metal-oxide-semiconductor FET (MOSFET) integration process. The ultrathin body and dangling-bond-free surface make it challenging to form good contact and high-quality high-κ dielectric in the 2D transistors. The flexible assembly of different 2D materials has broadly inspired the innovation of new mechanism transistors, which allow the 2D transistor to break the theoretical performance boundary of the silicon MOSFETs. In summary, the introduction of 2D materials has brought both opportunities and challenges in the performance breakthrough of transistor technology.

## The ultrasmall gate-length transistor demonstration

**Figure 1:**
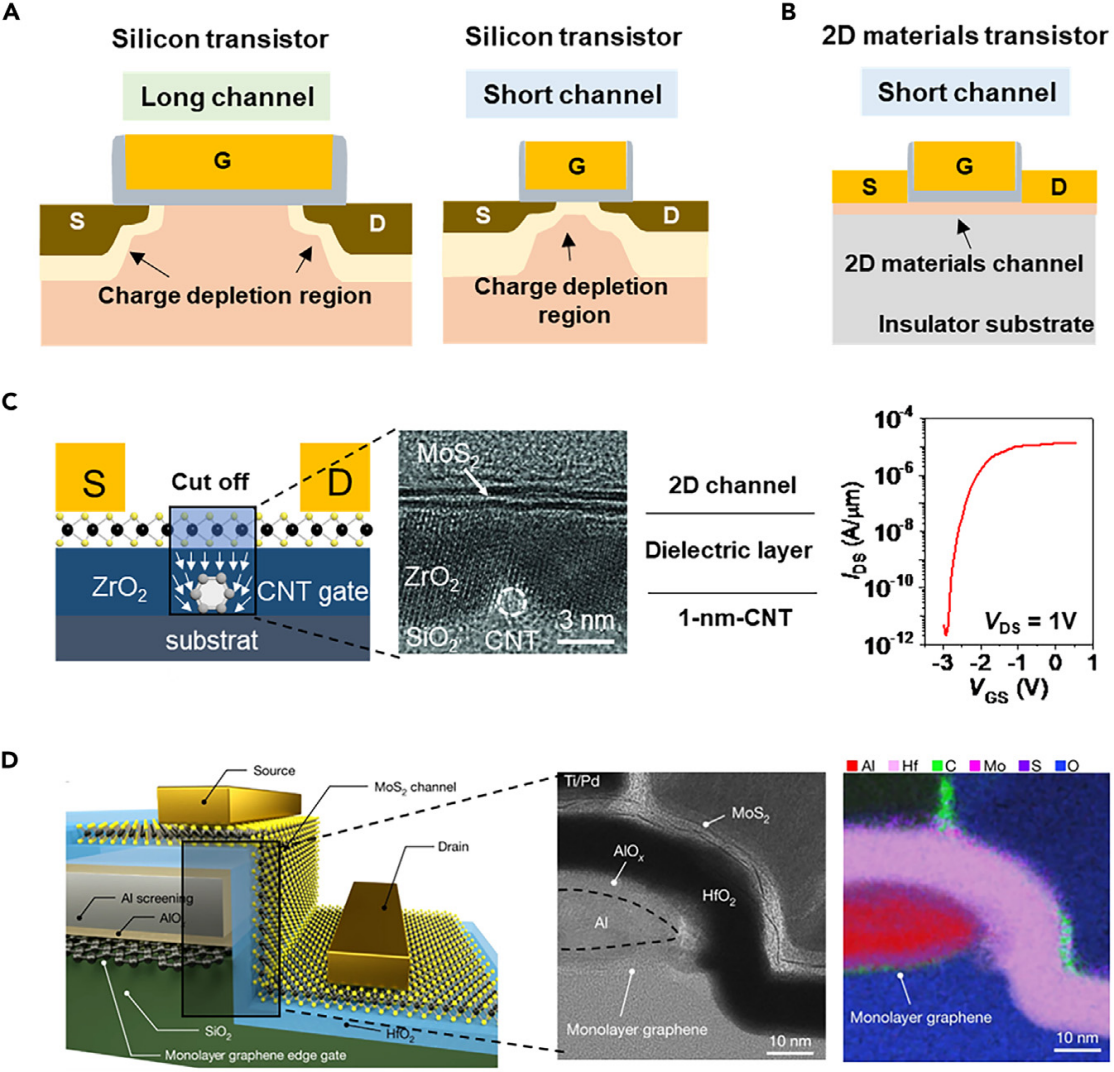
The ultrasmall gate-length transistor demonstration (A) The short channel effect in silicon transistors. (B) 2D transistors can be immune to the short-channel effect. (C) A demonstration of a 1 nm gate-length transistor. Reproduced with permission from (Desai et al., 2016) Copyright© 2016, American Association for the Advancement of Science. (D) A demonstration of graphene edge gated transistor. Reproduced with permission from (Wu et al., 2022) Copyright© 2022, Springer Nature.

It’s well-known that the performance of transistors can be continuously optimized according to a generalized scaling theory proposed by Dennard in 1974.^19^^,^^20^ According to this theory, the transistor’s lateral dimensions, transistor junction depth, oxide thickness and supply voltage are reduced by K^−1^ (K>1) and the substrate doping concentration is increased by a factor of K. Through the scaling down the size of the transistor, the chip density will increase by a factor of K^2^, and the device delay time and power consumption will reduce by a factor K^−1^ and K^−2^, respectively. However, for shorter channel devices, a series of short-channel effects arise that result in the failure of the theoretical prediction, such as the source/drain charge depletion region extending along the channel direction, and the drain-induced barrier lowering (Figure 1A). Although the FinFET technology has extended the technology node to “3 nm” (not the physical gate length of the transistor), the structure of the next-generation transistor has to change because the mobility of silicon FETs degrades quickly when the channel thickness is below 3 nm.^21^^,^^22^

Essentially, the scaling issues of silicon transistors are caused by the electrical performance degradation of the thin channel materials and the gate modulation being out of control to the short channel. The transistors with ultrathin 2D materials as channel material do not bother by these problems. The electrical properties of 2D materials will not degenerate even with a monolayer thickness. 2D materials, such as graphene, black phosphorus and transition-metal-dichalcogenides (TMDs), all have demonstrated good electrical properties with a very thin thickness. The graphene transistor is the first demonstrated 2D transistor and shows very high mobility but a limited on/off current ratio because of its zero bandgap.^23^^,^^24^ The black phosphorus transistor also shows high mobility and has an appropriate bandgap and is easily oxidized in the atmospheric environment.^25^^,^^26^ The TMDs have contained abundant materials and various electronic band structures, some of which show both large enough bandgap (1–2 eV) and good mobility (over 100 cm^−2^V^−1^s^−1^) for transistor applications,^27^^,^^28^^,^^29^^,^^30^^,^^31^ like the MoS_2_ and WSe_2_. As for the short-channel transistor demonstration, many works have experimentally proven that planar 2D transistors can be well worked with a very short channel length (less than 10 nm) which is beyond the ability of the planar silicon transistors.^32^^,^^33^ Because the ultrathin channel of the 2D transistor is on an insulator substrate, the carrier charge transportation is constrained within the 2D plane and no charge depletion region can extend along the channel on an insulator substrate (Figure 1B).

Particularly, some works have skillfully demonstrated that the gate length of a 2D transistor can be scaled below 1 nm. Desai et al.^34^ have fabricated a 1-nm-gate-length MoS_2_ transistor. In this work, a carbon nanotube (CNT, diameter of ∼1 nm) has been chosen as the gate terminal and ZrO_2_ serves as the high-κ dielectric. The 1 nm-length CNT was located by the scanning electron microscopy and optical microscope and then the bilayer MoS_2_ has been transferred above the CNT gate. The final device structure is shown in Figure 1C. It should be noted that the length between the source and drain metal contact is hundreds of nanometres, but the MoS_2_ that not controlled by the CNT can also serve as the source/drain interconnects because these parts’ MoS_2_ can be modulated to be good conductor by the voltage bias of silicon substrate. The ultrashort gate length device shows impressive electrical performance with a near-ideal SS of ∼65 mV/dec and a switching ratio of ∼10^6^. Except for using the CNT as an ultrashort gate, Jiang et al.^35^ have proposed a vertical-channel vdWs transistor in which the channel length can be controlled by the thickness of the deposited metal or dielectric materials. Subsequently, Wu et al.^36^ further developed this technology and demonstrated a vertical channel transistor with the edge of graphene as the side-wall gate. As Figure 1D has represented, the length of the gated vertical MoS_2_ channel is around 0.34 nm (the thickness of graphene is around 0.34 nm).

Besides the planar transistor, the multibridge channel (MBC) structure based on the gate-all-around (GAA) technology^37^^,^^38^^,^^39^ has been developed to realize the transistor with a feature size below 3 nm. For the MBCFET, 2D materials have provided a self-aligned edge-contact technology which paves the way toward higher-levels-stacked ultrathin MBCFET.^40^^,^^41^ In consideration of the ultrathin thickness of 2D materials, it is predicted that the channel material of MBCFET will switch to 2D materials when the technology node comes to 1 nm.^42^

## Alternative scaling route: Single logic transistor

Digital circuits contain massive logic gates and a basic logic gate needs several transistors to implement a logic function, for example, two transistors connected in series/parallel to realize the *AND*/*OR* logic functions. The smaller feature size of transistors can allow more computing cells to integrate into the chip. Alternatively, if the same logic functions can be carried out with fewer transistors, more functions will be integrated into the chip.

**Figure 2:**
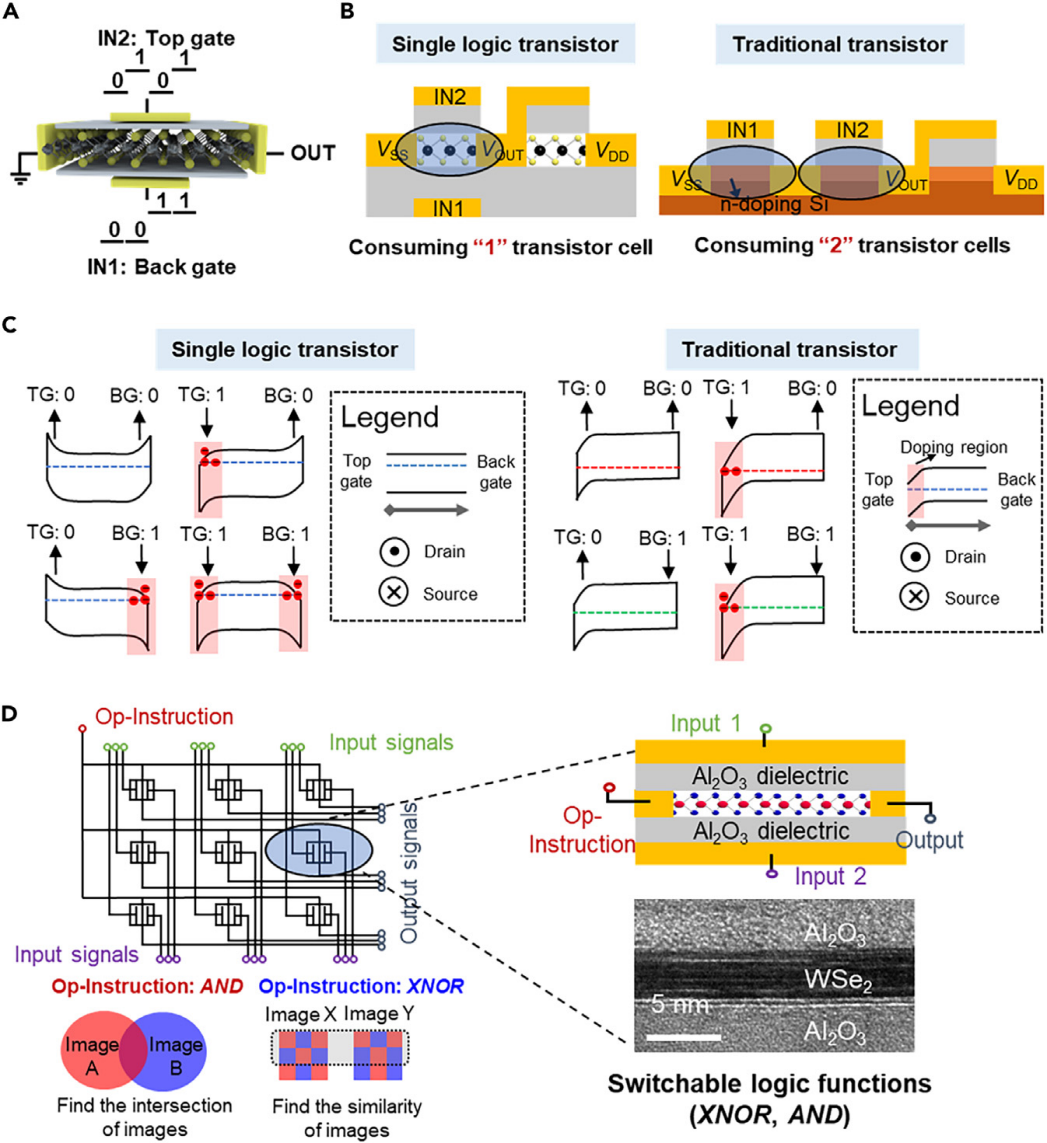
Alternative scaling route: single logic transistor (A) The schematic device structure of a single logic transistor. Reproduced with permission from (Liu et al., 2019) Copyright© 2019, Springer Nature. (B) The area efficiency advantage of the single logic transistor. Reproduced with permission from (Liu et al., 2020) Copyright© 2020, Springer Nature. (C) The mechanism comparison between the logic transistor and the traditional silicon transistor. Reproduced with permission from (Liu et al., 2019) Copyright© 2019, Springer Nature. (D) Logic function switchable single transistor and its image processing application. Reproduced with permission from (Zeng et al., 2022) Copyright© 2022, Springer Nature.

One promising solution is a reconfigurable logic device, where the devices’ function is not fixed and the same device can carry out different logic functions with various configurations.^43^^,^^44^^,^^45^^,^^46^ Compared with traditional logic gates, the reconfigurable logic device shows many more flexible logic functions. However, the function configuration usually needs additional area (e.g., additional regulate electrodes) which means the scaling down of a single reconfigurable device has more difficulty than that of the basic transistor structure. Liu et al.^47^ have experimentally demonstrated that a double-gated 2D transistor can realize logic computing (e.g., *AND*, *OR*) and there is no need for integrating additional regulated electrodes (Figure 2A). For a silicon transistor, only the top surface of the transistor can be regulated, therefore two top-gated transistors are needed to implement the two inputs logic gate (Figure 2B). In the single logic transistor, the top and bottom gates can regulate channel surfaces and serve as two input terminals. Figure 2C has comparing the mechanism difference between the single logic transistor and the traditional transistor. Subsequently, Zeng et al.^48^ further demonstrated that a single WSe_2_ double-gated transistor has multiple logic functions (*AND*, *XNOR*) and these functions are electrically switchable by the drain voltage. As Figure 2D has shown, using this reconfigurable single logic transistor as the pixel processing unit, the image processing array can perform two different image tasks and the consumption of transistors of this image processing unit is less than 16% of traditional circuits. A series of works have demonstrated the logic functions of this single logic transistor can be reconfigurable by other factors, such as photo illumination, working temperature.^49^^,^^50^

## The contacts optimization of 2D transistors

**Figure 3:**
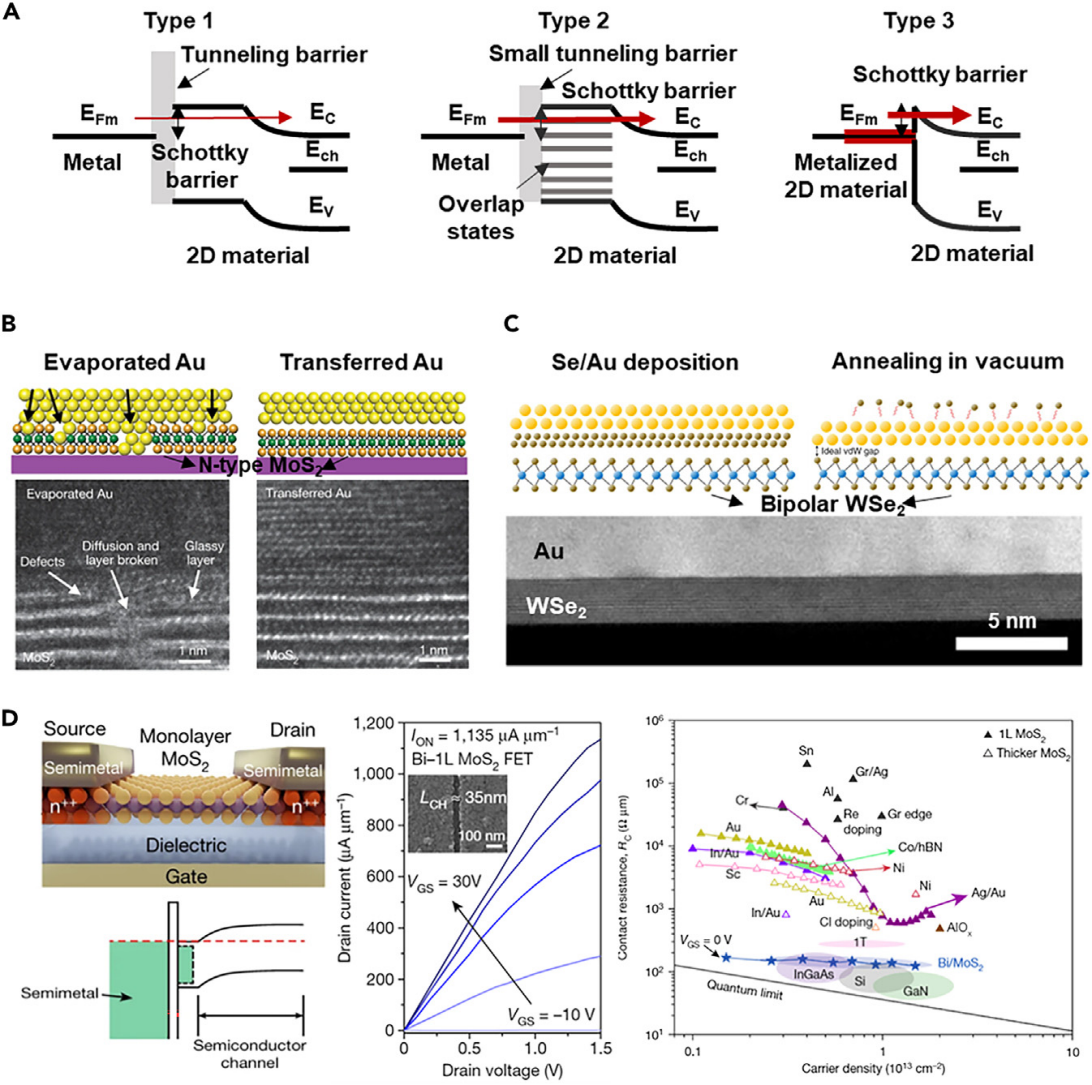
The contacts optimization of 2D transistors (A) The three types of metal-2D material junction contacts. Reproduced with permission from (Kang et al., 2014) Copyright© 2014, American Physical Society. (B) vdWs metal-2D materials junction formed by a transfer method. Reproduced with permission from (Liu et al., 2018) Copyright© 2018, Springer Nature. (C) The interaction- and defect-free vdWs metal-2D materials junction formed by a metal deposition process that uses a selenium buffer layer. Reproduced with permission from (Kwon et al., 2022) Copyright© 2022, Springer Nature. (D) Ultralow contact resistance between semimetal and monolayer semiconductors. Reproduced with permission from (Shen et al., 2021) Copyright© 2021, Springer Nature.

In the silicon MOSFET fabrication process, ideal nonrectifying contacts can be formed by heavy doping and silicon-metal alloy technology. When it comes to atomic thin film 2D materials with dangling bond free surface, there is still no good method for non-destructive and stable doping of few layer 2D materials. Most doping method of 2D materials is based on a solution or gas atmosphere, and the doping process is not compatible with large-scale integration.^51^^,^^52^^,^^53^^,^^54^ The unique layered structure of 2D materials raises challenges in the formation of low-resistance contact. Three types of metal-2D semiconductor contact are predicted by *ab initio* density-functional theory calculations, covering type 1, metals with very weak adhesion with 2D materials; type 2, medium adhesion; and type 3, strong adhesion.^55^^,^^56^ In type 1, there is a large Schottky barrier because the metal and the 2D materials do not contact well; in type 2, the tunnel barrier is negligible but there are many overlap states; in type 3, metal atoms strongly perturb the band structure of 2D materials and results in the metalized 2D materials (Figure 3A). Although the theory in the early stage has predicted that type 3 could form a stable and low-resistance alloy contact, the experimental results are not satisfying.

In the 2D transistor fabrication process, the metal-2D semiconductor junctions are chosen to modulate the contact performance. In the early studies, researchers generally believe that the Schottky barrier height is the key factor that domains the contact resistance of metal-2D semiconductors and a smaller barrier can guarantee better contact. The height of the Schottky barrier is the absolute difference between the work function of metal and the electron affinity (ionization potential) of the n-type (p-type) 2D materials. There is plenty of research showing that the 2D transistors can be modulated to behave in different polarity by tuning the contact metal work function.^12^^,^^57^^,^^58^ Chuang et al.^59^ even reported that the well-known n-type MoS_2_ transistor can be modulated to p-type when high work function materials (MoO_x_) are chosen as contacts. However, the MoS_2_ in this work is very thick (tens of nanometers) and typically the monolayer MoS_2_ with high work function metal contact materials is still n-type. For most monolayer 2D material transistors with contact metal deposited by the physical vapor deposition method, the Schottky barrier height seems pinned to a fixed position near the semiconductor bandgap and not sensitive to the work function of the contact metal.

Further studies show that the metal deposition process will damage the few-layer materials and lead to the Fermi-level pinning effect. Liu et al.^60^ have developed a metal transfer technology to form damage-free metal-2D materials junctions and Figure 3B has shown the good contact interface that formed by this transfer technology. In their work, the metal deposition damage issue has been solved and the transfer characteristics of transistors with monolayer channels can still be effectively tuned by the work function of transferred metal materials. The metal-transferred technology has effectively avoided the Fermi-level pinning effect, but the transferred method has a very low alignment precision which is not suitable for high-density integration. Kwon et al.^61^ have proposed another defect-free metal-2D material contact integration method that supports patterned metal deposition. A 10-nm-thick selenium layer was deposited by thermal evaporation in a high vacuum (<10^−9^ torr) and this process shows no damage to the surface of 2D materials. Subsequently, the contact metal (e.g., golden) can be deposited by electron-beam evaporation and the selenium buffer layer can be removed by annealing at 150°C in a high vacuum (<10^−9^ torr). As Figure 3C has shown, intimate and defect-free contacts can be formed and the transfer characteristics of 2D transistors can also be effectively tuned by the work function of deposited metal materials.

Although the Fermi-level pinning effect has been decreased by the innovative construction methods of metal-2D materials junction, the reported drive current and the contact resistance of the 2D transistors are still not satisfying. Shen et al.^62^ reported a semimetal-2D materials contacts technology in 2021, which shows impressive ultralow contact resistance that is close to the quantum limits (Figure 3D). Several other groups have quickly verified this technology and all of them reported improved device performance.^63^^,^^64^ In the first reported work, semimetal bismuth has been used as contacts on several 2D semiconductors, covering MoS_2_, WS_2_ and WSe_2_. Utilizing the semimetal as the contact, the metal-induced gap states are sufficiently suppressed and degenerated states are formed in the contact region. The bismuth-contacted monolayer MoS_2_ transistor has demonstrated a contact resistance of 123 Ω μm and drive current up to 1135 μA/μm.

Besides the geometry of the top contacts, the 2D transistor has developed a unique edge contact geometry.^40^^,^^65^^,^^66^ Surprisingly, 1D edge contact is enough to provide good contact and transmit a large drive current, which means the contact area of the 2D transistor will not be a limitation in ultrasmall feature size.

## The integration of ultrathin HIGH-κ dielectric on 2D materials

**Figure 4:**
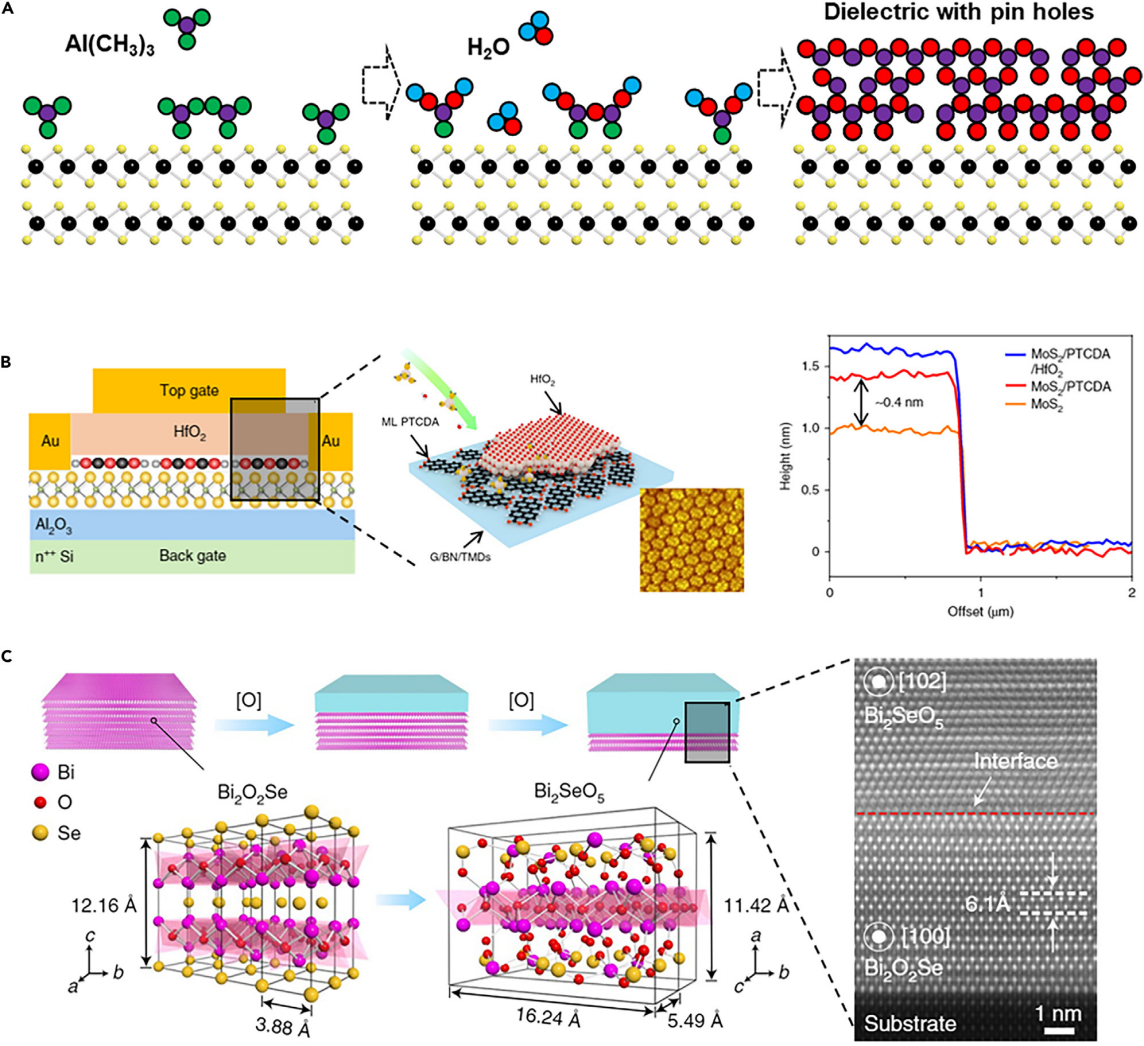
The integration of ultrathin high-κ dielectric on 2D materials (A) The deposition process of Al_2_O_3_ on 2D materials by ALD. (B) The uniform and ultrathin high-κ gate dielectrics formed by ALD that uses monolayer PTCDA as buffer layer. Reproduced with permission from (Li et al., 2019) Copyright© 2019, Springer Nature. (C) A native high-κ dielectric integration on 2D materials. Reproduced with permission from (Li et al., 2020) Copyright© 2020, Springer Nature.

The dangling-bond-free surface of 2D materials not only impacts the quality of the contact but also leads to the dielectric integration challenge. In the state-of-the-art integration circuit fabrication process, the high-κ gate oxide is deposited by the atomic layer deposition (ALD) technology and the small equivalent oxide thickness (EOT) of high-κ dielectric will advance the transistor performance. The ALD is a gas-phase chemical process and two precursors react with the surface of the channel material one at a time in a sequential and self-limiting manner. Therefore, the absence of dangling bonds will make the precursors hard to attach to the 2D materials. Figure 4A has briefly represented a Al_2_O_3_ deposition process by ALD on 2D materials. Typically, the directly deposited dielectric by ALD on a 2D material has a rough surface and pin-holes within the dielectric. Although there are some 2D dielectric materials, like hBN, that can directly transfer on the channel as good dielectric, the relatively small dielectric constant and the challenge in patterning dielectric make them not suitable for high-density integration. Integrating ultrathin high-κ dielectric on 2D materials is important for reducing power consumption and elevating the stability of the 2D transistors.

To improve the absorption of precursors on the dangling-bond-free surface, pretreatment of the surface of the 2D material is necessary. In the early stage, there are various pretreatment schemes to improve the quality of ALD high-κ dielectric on 2D materials, such as ultraviolet ozone and oxygen plasma. These methods will destroy the top layers of the channel materials and are not suitable for 2D materials with few layers. To avoid the damage of pretreatment to the channel, a remote oxygen plasma retreatment is developed where the ultralow energy plasma is physically adsorbed on MoS_2_ surfaces without making the flakes oxidise.^67^ But the absorption of the remotely flowed oxygen plasma cannot be well controlled. In general, moderate damage to the perfect surface of the 2D materials can make the dielectric integration easier but these methods are not suitable for the few-layer 2D materials. To stably realize large-scale sub-10 nm high-κ dielectric integration, an ultrathin metal (metal oxide) seeding layer method has been proposed.^68^ Before the ALD high-κ dielectric deposition, an ultrathin metal (∼1 nm) seeding layer is deposited on the channel material by physical vapor deposition. These metals should be easy to oxide (e.g. aluminum, yttrium) and after the oxidation, the high-κ dielectric can be deposited by a standard ALD process. Alternatively, adopting metal oxide as the seeding layer is also working well.^69^

In consideration that the quality of the metal oxidation buffer layer is not as good as the high-κ dielectric grown by ALD, the existence of the buffer layer makes the thickness of the dielectric hardly scale below 3 nm. Li et al. have explored a monolayer molecular crystal 3,4,9,10-perylenetetracarboxylic dianhydride (PTCDA) (∼0.3 nm) as the seeding layer which can allow the HfO_2_ scale below 3 nm and the EOT of the dielectric stack can be as low as 1 nm.^70^ A highly crystalline and uniform monolayer PTCDA can be self-limited epitaxial grown on the 2D materials and the carbonyl functional groups in PTCDA can function as sites for ALD nucleation and growth (Figure 4B). In consideration that the monolayer PTCDA is a low-κ material, further scaling the EOT of the dielectric stack below 1 nm will be challenging. Li et al.^71^ have discovered that the 2D materials Bi_2_O_2_Se can be layer-by-layer oxidized to form an atomically thin gate dielectric (Bi_2_SeO_5_). As Figure 4C has shown, similar to the Si/SiO2, the Bi_2_O_2_Se/Bi_2_SeO_5_ has a very good interface and the native oxide Bi_2_SeO_5_ can be also selectively etched away. Because the dielectric constant of Bi_2_SeO_5_ is around 21, this new technology can integrate a high-κ dielectric with an EOT scale to 0.9 nm. Subsequently, Zhang et al.^72^ have further optimized this technology and realized the selective oxidation through a lithography-compatible ultraviolet-assisted intercalative oxidation of the Bi_2_O_2_Se. The EOT of the dielectric Bi_2_SeO_5_ on the Bi_2_O_2_Se has also successfully decreased to 0.5 nm.

## The steep SS 2D transistors

**Figure 5:**
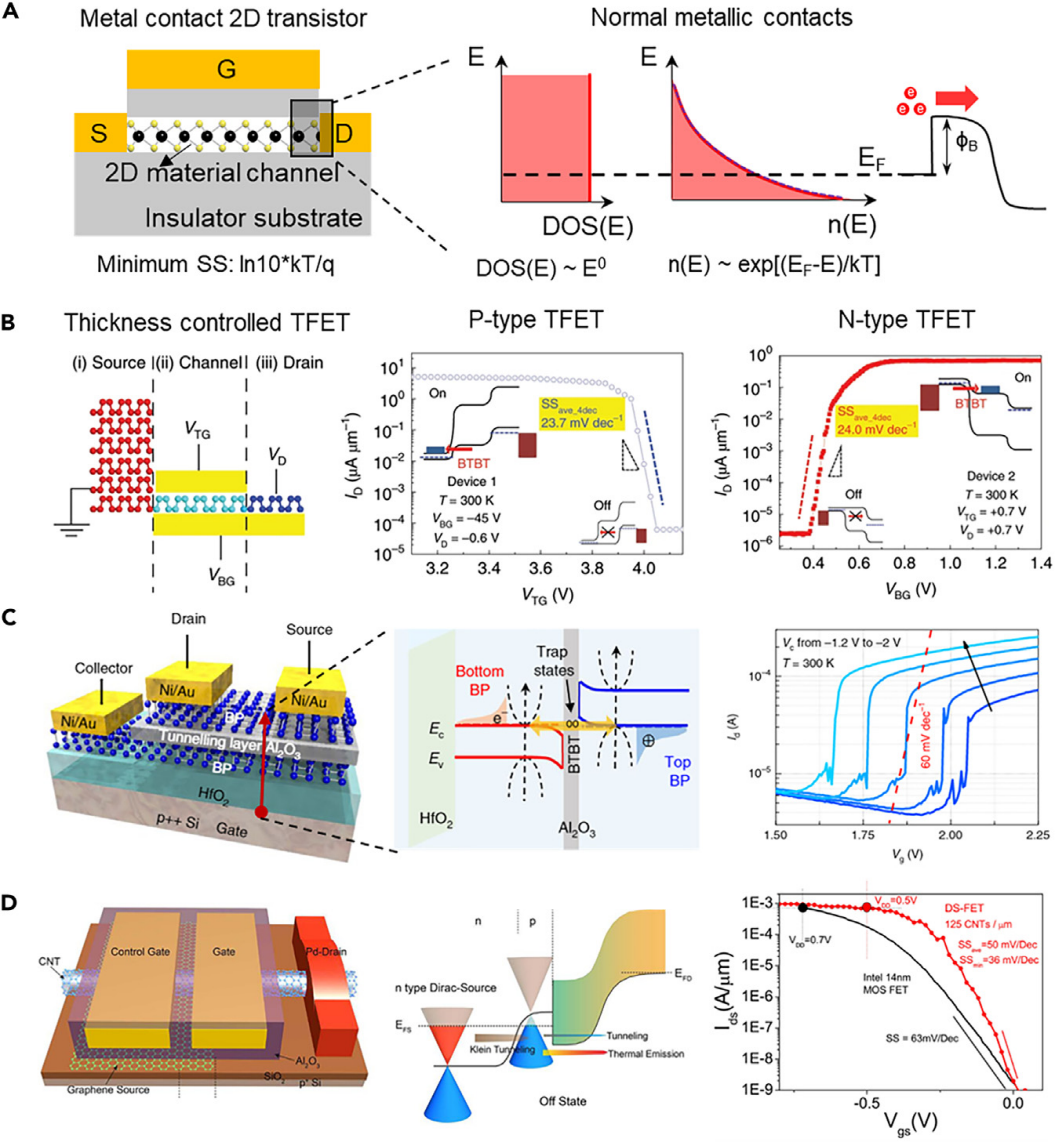
The steep SS 2D transistors (A) The minimum SS limitation in the metal contacts 2D transistor. (B) A thickness modulated black phosphorus TFET. Reproduced with permission from (Kim et al., 2020) Copyright© 2020, Springer Nature. (C) A transverse tunneling FET where the tunneling process occurs between the floating-gate and the channel. Reproduced with permission from (Xiong et al., 2020) Copyright© 2020, Springer Nature. (D) A Dirac-source FET with graphene as source electrode to provide an average SS of 40 mV/dec over four decades of current at room temperature. Reproduced with permission from (Qiu et al., 2018) Copyright© 2018, Springer Nature.

Besides the scale-down issue of the transistor feature size, the scaling of the supply voltage is also a challenge in the advanced technology node. In a conventional silicon MOSFET, the potential barrier of the source/channel is regulated by the gate voltage and the electron energy distribution of silicon (thermal Boltzmann distribution) makes the SS of the transistor cannot be below 60 mV/dec at room temperature. Limited by the minimum SS value, the supply voltage shrink-down is stagnant and the leakage current of an ultrathin dielectric becomes unacceptable.^73^^,^^74^ For the 2D transistor, this problem is also existing because the electron energy distribution of most 2D semiconductors is still Boltzmann distribution (Figure 5A). To break this minimum SS limitation, several new FET mechanisms have been researched, such as tunneling FET (TFET)^73^^,^^74^ and negative capacitance FET (NCFET).^75^ Compared with traditional bulk materials, 2D materials have strong advantages in the innovation of new mechanisms. The abundant 2D materials have provided rich electronic band structures which can provide more design space from band engineering. The flexible vdWs heterostructure assembling can also guarantee the designed band structure can be realized. The outbreak of various new steep SS mechanisms 2D transistor technologies has demonstrated the value of 2D materials and vdWs heterostructures.

TFET is one of the alternatives to the traditional MOSFET, the device mechanism is a band-to-band tunneling process where the hot electrons in the Fermi tail can be blocked by the bandgap. In a silicon TFET, the band alignment is realized by the different doping levels of the source, channel and drain region. Although 2D materials have enables more possibilities. Utilizing heterostructures can construct reasonable band structures, but the situation of interface could be challenging. The electronic band structures of 2D materials can be modulated by their thickness, for example, the bandgap of black phosphorus is varying from 0.3 eV (bulk) to 2 eV (monolayer). Kim et al.^76^ have reported a natural homogeneous black phosphorus TFET based on this bandgap modulation effect and there are no interface issues. Single black phosphorus with both bulk and monolayer regions was chosen to fabricate TFET, the bulk region functions as the source and the monolayer region covered with hBN and graphite functions as the channel and drain, respectively (Figure 5B). Both the n-type and p-type modes can realize average SS values over 4 decades of currents below 25 mV/dec at room temperature. There are also 2D-material-based NCFETs but they also have a hysteresis issue.^77^^,^^78^^,^^79^

Except for the steep SS mechanisms that already realized by the traditional bulk materials system, there are new mechanisms based on 2D materials have been proposed. Xiong et al.^80^ have proposed a transverse tunneling FET mechanism, which is not the same as the TFET. As Figure 5C has shown, the band-to-band tunneling in a TFET is occurring in the direction along the channel, whereas the tunneling current in the transverse tunneling FET is perpendicular to the channel. Although this transverse tunneling current made the SS could below 60 mV/dec, the leakage current (several microamperes) of the channel is not satisfying. Utilizing the unique Dirac point in the electronic band structure of graphene, Qiu et al.^81^ have put forward a new mechanism Dirac-source FET based on the graphene/CNT heterostructure. Different from the TFET, Dirac-source FET uses the Dirac point to cut off the Fermi tail to prove that a small SS and thermal emission at the graphene/CNT interface guarantee a large driving current (Figure 5D). For now, this is the most successful steep SS mechanism that showed the greatest balance in performance for both a small SS and a large driving current. This new mechanism is also verified in the graphene/MoS_2_ heterostructure.^82^

## The ultrafast floating-gate transistor

**Figure 6:**
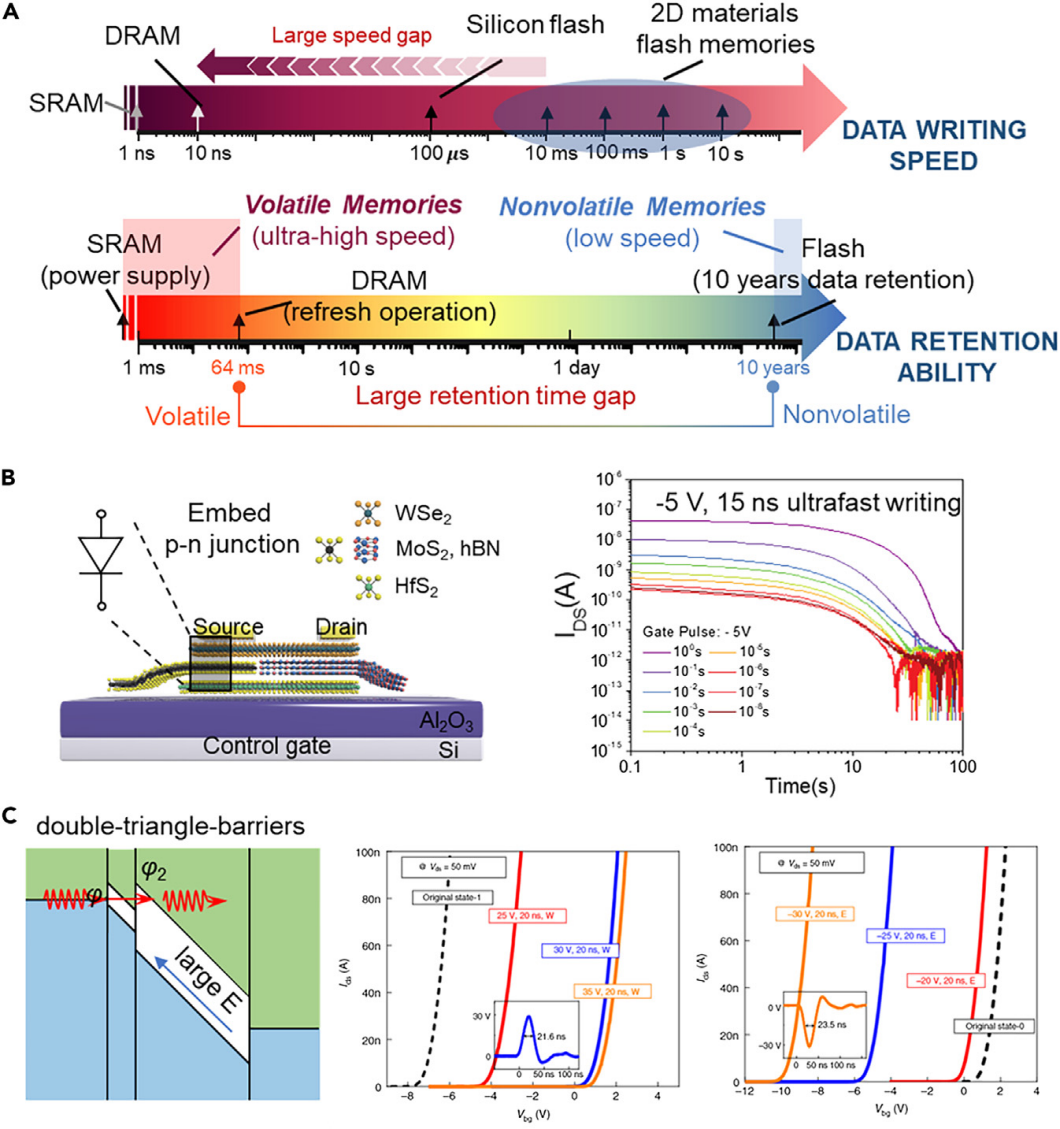
The ultrafast floating-gate transistor (A) The data writing speed and retention ability trade-off among the volatile and non-volatile memories. Reproduced with permission from (Liu et al., 2018) Copyright© 2018, Springer Nature. (B) A semi-floating gate transistor for ultrafast data writing applications. Reproduced with permission from (Liu et al., 2018) Copyright© 2018, Springer Nature. (C) Ultrafast non-volatile floating gate transistor enabled by vdWs heterostructure design. Reproduced with permission from (Liu et al., 2021) Copyright© 2021, Springer Nature.

In integrated circuits, the transistor is not only functions as a switch but also used to store data. The mainstream memory technologies are all based on transistor structure. The high-speed memories are volatile memory, covering static random access memory (SRAM) and dynamic random access memory (DRAM). Both of them are based on transistor technology and have limited volume because of their complex structures, SRAM is built with six transistors and DRAM has a 1 transistor-1 capacitor structure. Flash memory is representative of high-density non-volatile memory (data can be stored for more than 10 years) and its basic structure is a simple floating-gate transistor where a floating gate is inserted into the gate dielectric functions as the charge storage. Figure 6A has indicated that the big challenge in all these charge memory technologies is the contradiction between the programming speed and the charge storage ability. The volatile memories have ultrafast programming speed (SRAM∼1 ns, DRAM∼10 ns) but the data only maintains while the device is powered and their storage volume is also limited. Non-volatile memory has 10 years of data storage ability without a power supply and is high-density, but the accessing speed is limited to ∼100 μ s.^83^ The floating-gate transistor support high-density integration and the programming speed breakthrough of a floating-gate transistor is important.

There are plenty of studies that use 2D materials to fabricate floating-gate transistors. In the prior studies, the 2D materials were mainly used as channel and floating gate materials.^84^^,^^85^^,^^86^^,^^87^^,^^88^^,^^89^^,^^90^^,^^91^^,^^92^^,^^93^^,^^94^^,^^95^ Thinner channel materials can achieve a larger memory window and the thinner floating-gate layer will reduce the crosstalk between different cells. However, the programming speed of these 2D floating-gate transistors is limited to the millisecond, far below the speed of volatile memory. The strength of 2D floating-gate transistors is that their band structure can be flexibly designed and the band engineering can be used to regulate the electrical performance of memory devices.^96^ Liu et al.^97^ have developed an attractive semi-floating gate (SFG) memory technology that largely advances the device programming speed to the level of DRAM. Figure 6B shows the structure of 2D SFG memory and there is a WSe_2_/MoS_2_ p-*n*-heterojunction has been embedded into the tunneling layer. Utilizing the embedded p-*n*-junction, the charges of the channel material can flow into the floating gate using relatively small voltage control with an ultrahigh-speed writing operation (−5 V, 15 ns). However, the introduction of the p-*n*-junction brings about an ultrafast speed but also causes the data retention time (10s) is not long enough because of the charge leakage. Although the retention time can be enhanced by local field modulation (63.5 s)^98^ and much longer than that of DRAM (64 ms), an ultrafast non-volatile floating-gate transistor would be desirable.

In 2021, Wu et al.^99^ and Liu et al.^100^ independently published an ultrafast non-volatile floating-gate transistor using vdWs heterostructure. Wu et al. owed the extraordinary programming speed to the atomically sharp interfaces and demonstrated that the data can be stored for at least 5400 s without noteworthy degradation (linear extrapolation: 10 years retention time). Liu et al. have further verified the ultrafast non-volatile characteristics in a high-temperature test (commercial standard retention time test). Besides the strengthening of a suitable barrier height, gate coupling ratio and clean interface, Liu et al. also indicated that the tunneling mechanism of the 2D floating-gate transistor is not the same as the traditional silicon floating-gate transistor. As shown in Figure 6C, the energy bands of the channel can be sharply bent because of ultrathin electrostatic modulation of 2D semiconductors, which forms double barriers to enhance tunneling efficiency. Although the programming speed has dramatically improved, the ultrafast non-volatile transistors are based on high operation voltage and the thickness of the memory stack should be further optimized to cut down the amplitude of the programming.

## Conclusion and outlook

Making use of the ultrathin body and the dangling-bond-free surface, 2D materials demonstrate remarkable advantages in size downscaling and heterostructure innovation. The ultrashort gate-length transistor demonstration is far beyond the ability of the planar silicon transistor. The various steep SS transistors and ultrafast floating-gate transistors have significantly broken the performance limitations of silicon transistors. However, the perfect interface also leads to issues on large-scale integration. There is already huge progress in the integration process, because we have discussed in the contact optimization and dielectric integration parts, but the large-scale integration process still needs heavy study. In the following, we will briefly discuss the challenges for the application of the 2D material from lab to fab.

For large-scale integration, wafer-scale growth of 2D materials is essential. For now, there are only a few kinds of 2D materials that can be large-scale grown and accurately controlling the phase and layer number of the 2D materials remains a challenge.^101^^,^^102^ A stable and effective doping method of 2D materials is necessary for building energy-efficient complementary circuits but this field is rather slow in progress. The material growth with proper doping is still the key obstacle that hinders the 2D transistor from lab to fab. As for the device fabrication, the contacts and dielectric integration are in the first place to guarantee the performance of integrated circuits. Although the bismuth contacts provide a low enough contact resistance on 2D materials (123Ω μm), similar to the contact resistance in the state-of-the-art silicon transistor, the melting point of bismuth is too small to be compatible with the CMOS process. More semimetal contact schemes with higher temperature tolerance should be explored, such as antimony.^103^ The dielectric integration on 2D materials has challenges to scaling the EOT below 1 nm and the high-κ deposition process will also affect the threshold voltage of channel.^104^ The self-oxidized native dielectric can realize EOT to 0.5 nm, but the applicative materials are very limited.

What’s more, a large portion of high-performance 2D transistors is based on the vdWs heterostructure which brings forward great challenges in large-scale integration. The vdWs integration method can allow flexible assemble various heterostructures, but the transfer precision is very poor and it is still no way to improve the precision to that of lithography. To integrate heterostructure devices with high density, selective etching 2D materials^105^^,^^106^^,^^107^ and self-aligned process^40^^,^^108^ should be developed.
